# Return-to-work intervention for cancer survivors: budget impact and allocation of costs and returns in the Netherlands and six major EU-countries

**DOI:** 10.1186/s12885-015-1912-7

**Published:** 2015-11-12

**Authors:** Janne C. Mewes, Lotte M. G. Steuten, Iris F. Groeneveld, Angela G. E. M. de Boer, Monique H. W. Frings-Dresen, Maarten J. IJzerman, Wim H. van Harten

**Affiliations:** 1 Health Technology and Services Research, Faculty of Behavioural, Management and Social Sciences, University of Twente, PO Box 217, 7500 AE Enschede, The Netherlands; 2 Hutchinson Institute for Cancer Outcomes Research, University of Washington, 1110 Fairview Avenue North, 98109 WA Seattle, USA; 3 Rijnlands Rehabilitation Centre, Wassenaarseweg 501, 2333 AL Leiden, The Netherlands; 4 Sophia Rehabilitation, Vrederustlaan 180, 2543 SW The Hague, The Netherlands; 5 Coronel Institute of Occupational Health, Amsterdam Medical Center, Meibergdreef 9, 1105 AZ Amsterdam, The Netherlands; 6 Department of Psychosocial Research and Epidemiology, Netherlands Cancer Institute, Plesmanlaan 121, 1066 CX Amsterdam, The Netherlands

**Keywords:** Budget impact analysis, Return-to-work, Counselling, Exercise, Cancer survivors, Financial incentives

## Abstract

**Background:**

Return-to-work (RTW)-interventions support cancer survivors in resuming work, but come at additional healthcare costs. The objective of this study was to assess the budget impact of a RTW-intervention, consisting of counselling sessions with an occupational physician and an exercise-programme. The secondary objective was to explore how the costs of RTW-interventions and its financial revenues are allocated among the involved stakeholders in several EU-countries.

**Methods:**

The budget impact (BI) of a RTW-intervention versus usual care was analysed yearly for 2015–2020 from a Dutch societal- and from the perspective of a large cancer centre. The allocation of the expected costs and financial benefits for each of the stakeholders involved was compared between the Netherlands, Belgium, England, France, Germany, Italy, and Sweden.

**Results:**

The average intervention costs in this case were €1,519/patient. The BI for the Netherlands was €-14.7 m in 2015, rising to €-71.1 m in 2020, thus the intervention is cost-saving as the productivity benefits outweigh the intervention costs. For cancer centres the BI amounts to €293 k in 2015, increasing to €1.1 m in 2020. Across European countries, we observed differences regarding the extent to which stakeholders either invest or receive a share of the benefits from offering a RTW-intervention.

**Conclusion:**

The RTW-intervention is cost-saving from a societal perspective. Yet, the total intervention costs are considerable and, in many European countries, mainly covered by care providers that are not sufficiently reimbursed.

## Background

Many cancer survivors experience difficulties in returning to work. Approximately 40 % have not resumed work 24 months post treatment [1]. Furthermore, cancer survivors have an increased risk for unemployment compared to the general population [2, 3]. Supporting patients in returning to the workplace may improve health and quality of life, and avoid high societal costs associated with unemployment and long-term inability to work [4–6]. When successful, RTW-interventions can increase productivity through reducing sick leave and might save costs to society. However, RTW is not or only partly reimbursed by health insurers in most European countries, including the Netherlands. The main reason is that the interventions are expected to be expensive and unaffordable. However, when they are effective, return-to-work interventions can produce financial benefits, although their size is unknown. To date, no budget impact analysis of these interventions or of any other cancer rehabilitation intervention has been published. Evidence on the budget impact would quantify to what extent return-to-work interventions are beneficial from a financial point of view or if these interventions would add costs to the system. Therefore, the primary aim of this study was to evaluate the expected budget impact (BI) of RTW-interventions for cancer survivors. In a budget impact analysis, the expected financial impact of an intervention on the budget of a health system is analysed [7].

Return-to-work interventions typically consist of counselling by an occupational physician directed on return-to-work possibilities. We considered an intervention that combines counselling with physical exercise, as a Cochrane review showed that multidisciplinary RTW-interventions are most effective. Moreover, physical exercise is strongly recommended for cancer patients in several organisations’ guidelines [8–12]. The analysis was conducted from the Dutch societal perspective and from the perspective of a hypothetical cancer centre over the time period 2015–2020. The latter perspective serves to estimate the BI for cancer centres that plan to introduce RTW and want to investigate its year-by-year financial impact. The secondary objective was to identify the allocation of costs and financial returns of providing RTW for cancer survivors for several European countries. This provides insights in financial incentives for and against RTW-implementation.

## Methods

### Budget impact analysis

The budget impact of a multidisciplinary RTW-intervention was assessed following the International Society for Pharmacoeconomics and Outcome Research-guidelines over a time horizon of 5 years. We compared the situation in which the intervention gradually is implemented to current practice, where only 5 % of the eligible patients can follow the intervention. We considered a Dutch societal perspective and that of a cancer centre serving a population of 1 m inhabitants, which equals the catchment area of large European cancer centres [7, 13]. All input parameters are presented in Table 1.

**Table 1:** Input parameters for the budget impact analysis

Parameter	Value for the Netherlands	Value for a reference cancer centre	Source
Cancer incidence in the Netherlands:			
2015	110,215	6,533	Signaleringscommissie Kanker of the Dutch Cancer Society, 2011 [4]
2016	112,776	6,675	
2017	115,337	6,816	
2018	117,899	6,805	
2019	120,460	7,097	
2020	123,021	6,237	
Percentage of eligible patients:			
% with 5-year survival	62 %		Dutch Cancer Registry [15]
% aged 25–64	40 %		Dutch Cancer Registry [14]
% with treatment outcome that allows	80 %		Assumption
RTW			
% who want to resume work	85 %		Assumption
% who wish to follow intervention	70 %		Assumption
% eligible for intervention	12 % (=1*0.32*0.80*0.85*0.70)		Product of the above
Capacity in current practice:			
2015	5 %	5 %	Assumptions
2015	5 %	5 %	
2016	5 %	5 %	
2017	5 %	5 %	
2018	5 %	5 %	
2019	5 %	5 %	
2020	5 %	5 %	
Capacity in new situation:			
2015	20 %	30 %	Assumptions
2016	30 %	60 %	
2017	40 %	90 %	
2018	50 %	90 %	
2019	60 %	90 %	
2020	70 %	90 %	
Percentage of patients for whom the intervention is reimbursed in current situation:	100 %		
Percentage of patients for whom the intervention is reimbursed in new situation:	10 %		Assumption
Intervention costs	€ 1,517		Assessment of the costs based on intervention description [13] and information provided from the staff who delivered the intervention.
Additional weekly working hours in the new situation	5.8 h		Thijs et al., 2011 [17]

Approval of an ethics committee and the participants’ consent were not required for this research, as the data were derived from the literature and from health professionals. Dutch law does not require medical or ethical reviews for interviews with health care professionals. Confidentiality was ensured by not disclosing the names or hospitals of the interviewees and only referring to them by country. The study on which the cost calculation is based had received approval from the respective ethics committee [12].

#### Intervention description and uptake of the intervention

The RTW-intervention, including counselling and exercise, is prescribed at the start of cancer treatment to all cancer patients who potentially can and wish to resume work. The counselling includes two one-hour sessions with an occupational physician specialised in oncology. The exercise component consists of 24 group sessions of moderate to high-intensity physiotherapy in groups of five. The duration of the exercise programme is 12 weeks, starting at the onset of chemotherapy. Some hospitals also provide a sports medical capacity-assessment before and after the programme. A more detailed description of the intervention was published previously [12, 14].

#### Eligible population

Patients with any type of cancer are eligible when they are; a) of working age, i.e. between 25 and 64 years, b) treated with curative intent [4, 15, 16], c) expected to have a treatment outcome that allows returning to work, d) wishing to return to work, and e) willing to follow the intervention. For each criterion, the percentage of all cancer patients to whom this applies was analysed and is given in Table 1. The percentage of patients who are eligible was calculated by multiplying 100 % with the percentages of all criteria. This resulted in an eligibility percentage of 12 % of all cancer patients that are diagnosed yearly (see Table 1).

#### Capacity

As there currently is insufficient capacity for providing the intervention, not all patients eligible for a multidisciplinary return-to-work intervention can follow it. Reasons for the limited capacity are that the implementation in general is still in the starting phase and that many health professionals are not fully aware of the possibilities that cancer rehabilitation offers. In order to offer the intervention to all eligible patients and provide the intervention on a larger scale, hospitals would, e.g., first need to employ more physical therapists and occupational physicians, and create the appropriate organisational structures for providing the intervention on a larger scale.

As a result of the above, currently only a small (i.e. 5 %) subgroup of survivors is prioritized to receive multidisciplinary rehabilitation treatment. Thus, of the 12 % of the cancer patients who are eligible, 5 % can follow the intervention. This capacity is assumed to remain at that 5 % level throughout the analysis’ time horizon for current practice. This is compared to the situation in which hospitals start to implement the intervention and gradually increase the capacity to enrol patients, starting with 30 % of eligible patients in 2015–70 % in 2020. Thus, in 2015, 30 % of the 12 % of eligible survivors follow the intervention. Finally it is expected that in 2017 most of the eligible survivors (70 %) can participate in multidisciplinary return-to-work interventions.

The capacity in a single cancer centre that is used for the analysis from the perspective of a hypothetical cancer centre rises much faster, from 30 % in 2015–90 % in 2020. It is assumed that once a cancer centre decides to offer the intervention it would take measures to relatively quickly provide it to all eligible patients. However, they would also be faced by shortages of staff, especially occupational physicians. The percentage from the Dutch societal perspective remains lower, at 70 %, because it is assumed that not every hospital will offer the intervention. Thus, some hospitals will not offer return-to-work interventions at all, whereas some offer it to 90 % of the eligible patients, leading to an overall percentage of 70 %.

#### Costs of the intervention and impact on other costs

Intervention costs include staff costs, administration, materials, and 42 % overhead, according to the Dutch manual for cost research [17]. Volumes of resource use were obtained from the intervention protocol and health professionals participating in a feasibility study of the intervention [12, 14]. Unit costs were determined following Dutch guidelines for pharma-economic research [17]. Staff training costs of €335 were considered as part of the overhead. Hospitals receive reimbursement for providing RTW-interventions to patients formally indicated for multidisciplinary rehabilitation. This is circa 10 % of the eligible population. In current practice, all patients who receive the RTW-interventions are indicated for multidisciplinary rehabilitation and thus receive reimbursement.

An impact on other costs occurs through changes in the patients’ productivity. The effect of RTW on resuming work was taken from a Dutch trial. In the intervention group the participants followed an 18-weeks exercise programme, consisting of a high intensity resistance and endurance training. This was compared to standard medical care that was received by an age-matched control group. Patients with any type of cancer of 18–65 years of age were included who were treated with curative intend and were in paid employment at the time of diagnosis. 110 patients were included in the analysis, 72 in the intervention group and 38 in the control group. The adherence of the participants was very high with 96 % and thus slightly higher than for the multidisciplinary return-to-work intervention where it was 86 %. A significant difference in the time to resume work was not found. However, the intervention was found to increase productivity significantly by 5 · 8 h/week for one year [18]. Thus, the participants in the intervention group were able to work more in the long-term. This 5.8 h/week that are worked more than in current practice was used for the productivity benefit in this analysis and was €30.02/h, according to the Dutch manual for cost research [17].

#### Analysis

**Fig. 1:**
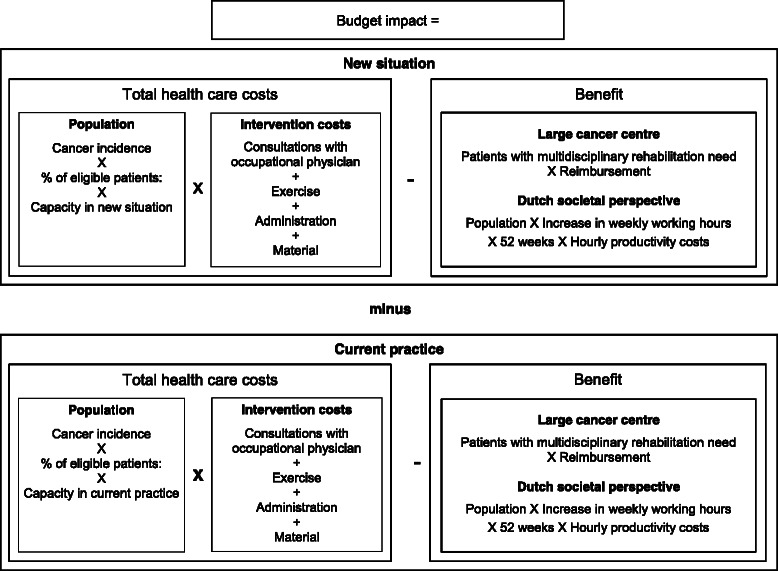
Structure of the budget impact model

**Fig. 2:**
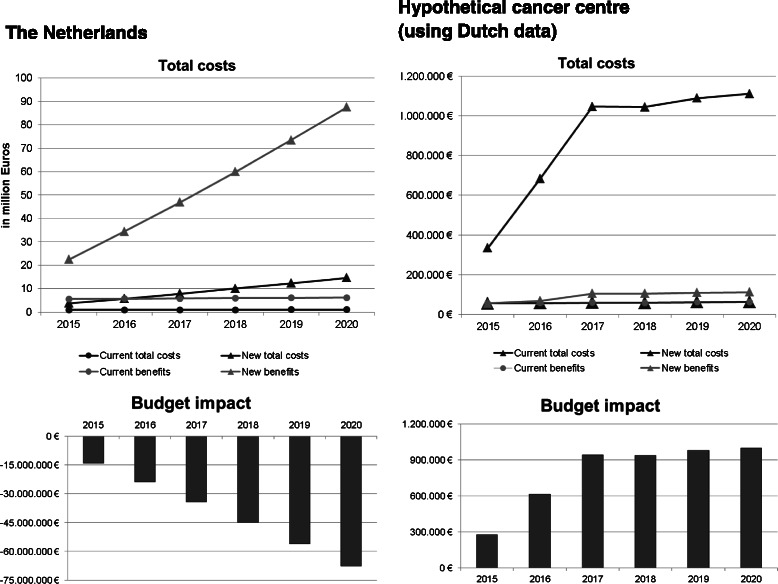
Results of the base case analysis. A negative budget impact indicates that the intervention is cost-saving. The positive budget impact for the cancer centre results from the situation that in the Netherlands, the costs for RTW are not reimbursed for most patients. Thus, if a hospital is offering the intervention they need to finance it themselves

For analysing the budget impact, a spreadsheet model (Fig. 1) was created in Microsoft Excel (Redmond, WA). The budget impact equals the total cost of the RTW-intervention minus the productivity gains that accrue from RTW, in the new situation vs. current practice [7]. In the model, the number of patients following the intervention was identified by multiplying cancer incidence with the percentage of eligible patients and the capacity of hospitals to provide the intervention. The number of patients was then multiplied with the intervention costs, which resulted in the total costs of the RTW-intervention. For the Dutch societal perspective, the productivity gains equal the number of patients who follow the intervention multiplied with the additional yearly working time generated and with the hourly productivity costs. For the cancer centre’s perspective, the benefit consists of receiving reimbursed from the health insurer for delivering RTW to the 10 % of the patients indicated for multidisciplinary rehabilitation Fig. 2.

#### Sensitivity analysis

The influence on the budget impact of the effectiveness of the intervention was analysed, in order to test the robustness of the model outcomes. For this purpose, the effect in a range of 0 to 5.8 additional weekly working hours, which corresponds to 0–302 h a year, was used to display its effect on the budget impact graphically. This also allowed analyzing where the budget impact changes from being cost-saving to adding costs, i.e. where the line of the budget impact crosses the x-axis from being cost-saving to adding costs.

### Analysis of the incentive structure to implement RTW in the Netherlands and in several EU-countries

The allocation of costs and financial returns across stakeholders involved in RTW was analysed, to identify potential (dis)incentives for implementing RTW-interventions. For this purpose, an email survey was conducted among comprehensive cancer centres that are members of the Organisation of European Cancer Institutes (*n* = 40) from the Netherlands, Belgium, England, France, Germany, Italy, and Sweden. At least one respondent from each country was required. In the survey each cancer centre-representative with professional knowledge about the healthcare and welfare system in their respective country, was asked to tick in a list of stakeholders which of these (1) bear the costs of sick leave of cancer patients, (2) are responsible for the reintegration of cancer patients into the workplace, (3) bear the costs for offering an RTW-intervention, and (4) benefit financially from cancer patients following a RTW intervention. The list of stakeholders included health insurers, hospitals, patients, employers, pension insurance schemes, and the state. The nature of the financial benefits depends on the stakeholder and includes, e.g., for hospital reimbursement by the patients’ health insurers, for health insurers a reduction in the patients’ future health care needs, for patients the ability to continue working and receive an income, or for employers the prevention of sick leave and subsequent production losses.

For the analysis, the results of the survey were assessed in 2*2 tables showing how many and which stakeholders both pay and gain from RTW, how many only pay or only gain, and how many do not pay or gain.

## Results

### Budget impact analysis

#### Base case results

The number of patients following the intervention under current (Dutch) practice on national level was estimated to be 651 in 2015 and increase to 726 patients in 2020. This increase only reflects the rising cancer incidence, while the percentage of patients who are eligible for the interventions remains stable in this model. In the new situation in which RTW-interventions are rolled out more widely, 2,602 patients would participate in the intervention in 2015. As cancer incidence and the capacity both rose, it is estimated that in 2020, 10,166 patients would follow the intervention.

The same reasoning as described above was applied to estimate the number of patients receiving RTW in a large cancer centre. In current practice this number would rise from 39–43 in the 5-year period in our model, due to the growth in cancer incidence. After implementing RTW, the number could increase from 231 in 2015–769 in 2020, as the capacity for treating the eligible patients is assumed to grow.

The average costs of the RTW-intervention are estimated at €1,517 per patient, of which €567 (37 %) are for consultations with the occupational physician, €879 (59 %) for the exercise part, and €46 for administration and printed materials.

The total health care costs when implementing RTW for the Netherlands are €4.0 m to €15.4 m from 2015–2020. The benefits in terms of productivity gains are €23.6 m to €92.0 m in 2015–2020. The BI for the Netherlands is €-14.7 m in 2015, rising to €-71.1 m in 2020, meaning that from a societal perspective RTW for cancer survivors in the Netherlands would be cost-saving. The productivity gains are large and outweigh the intervention costs by far. In fact, with rising incidence and a growing proportion of patients following RTW, cost savings further increase year-by-year. However, the intervention is rather expensive to its providers and the initial health care costs are considerable.

For a large cancer centre, the costs for the intervention compared to current practice, from 2015–2020 increase from €351 k to €1.2 m. The financial benefit in terms of reimbursement from 2015–2020 is only €58.6 k to €116.8 k. The BI for a cancer centre is €292.8 k in 2015 and rises to €1.1 m in 2020. Thus, for a cancer centre providing this service, the high intervention costs cause RTW to be an expensive intervention to offer, as they get only reimbursed for 10 % of the patient population. Table 2 and Fig. 2 show the base case results.

**Table 2:** Results of the budget impact analysis

	2015	2016	2017	2018	2019	2020
DUTCH SOCIETAL PERSPECTIVE						
Current practice:						
Number of patients	651	666	681	696	711	726
Health care costs	987.901 €	1.010.858 €	1.033.815 €	1.056.772 €	1.079.729 €	1.102.686 €
Productivity benefits	5.889.947 €	6.026.819 €	6.163.690 €	6.300.562 €	6.437.434 €	6.574.306 €
New situation:						
Number of patients	2.602	3.994	5.446	6.959	8.532	10.166
Health care costs	3.951.605 €	6.065.149 €	8.270.522 €	10.567.724 €	12.956.753 €	15.437.611 €
Productivity benefit	23.559.787 €	36.160.912 €	49.309.524 €	63.005.623 €	77.249.210 €	92.040.284 €
Budget impact	−14.706.137 €	−25.079.802 €	−35.909.126 €	−47.194.109 €	−58.934.752 €	−71.131.054 €
PERSPECTIVE OF A HYPOTHETICAL CANCER CENTRE						
Current practice:						
Number of patients	39	39	40	40	42	43
Health care costs	58.560 €	59.828 €	61.093 €	60.999 €	63.611 €	64.864 €
Benefits (reimbursement)	58.560 €	59.828 €	61.093 €	60.999 €	63.611 €	64.864 €
New situation:						
Number of patients	231	473	724	723	754	769
Health care costs	351.358 €	717.939 €	1.099.674 €	1.097.987 €	1.144.994 €	1.167.550 €
Benefit (reimbursement)	58.560 €^a^	71.794 €	109.967 €	109.799 €	114.499 €	116.755 €
Budget impact	292.798 €	646.145 €	989.706 €	988.188 €	1.030.495 €	1.050.795 €

^a^According to the model, this would be €35,136, assuming that hospitals receive reimbursement for 10 % of the patients. As this is lower than the benefit in the current situation, it is expected that as long as in the new situation there still are patients with a multidisciplinary rehabilitation need (for whom the costs are reimbursed by insurance), these would be treated preferentially to patients for whom the costs are not reimbursed

#### Sensitivity analysis

**Fig. 3:**
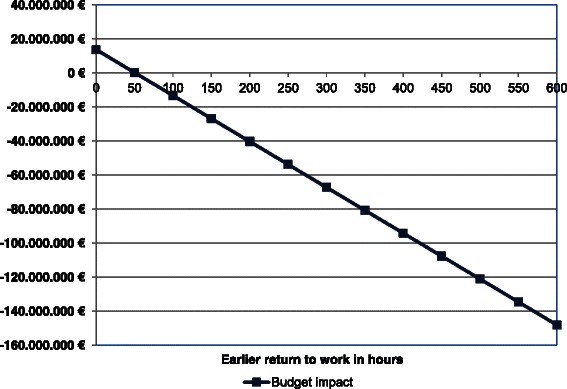
The impact of earlier RTW on the budget impact in 2020. From 2020 on a steady state is assumed

Figure 3 shows that even when the benefit of the RTW-intervention was much smaller than expected based on current data the intervention would still be cost-saving. The health care costs equal the productivity benefits, i.e. the BI is zero, when the RTW-intervention enables patients to return to work 50.6 h earlier in 2020 compared to usual care. This value corresponds to an increased weekly working time of approximately 1 h/week, which is more than five times lower than the value used in the base case analysis (5.8 h/week).

### Analysis of the incentive structure for implementing RTW

#### The Netherlands

Health care providers that offer RTW-interventions carry their costs themselves, apart from the health insurances’ reimbursement for about 10 % of patients with a multidisciplinary rehabilitation need. The financial returns from earlier RTW are received by employers, the patients, and pension funds. Thus, a misalignment exists between the stakeholders that pay for RTW and those that receive the financial benefits in terms of increased productivity or in preventing invalidity pension. As this situation discourages to offer RTW on a large scale, its substantial cost-savings to society are forgone. A cancer centre or hospital would need to highly value the intangible benefits, such as being a provider of high-quality care, attractiveness for patients, or being a leading cancer centre, in order to make up for the costs. See Table 3 for the results of this analysis.

**Table 3:** Distribution of RTW intervention costs and financial returns across the stakeholders for the Netherlands

	Health insurance	Hospitals/Health care providers	Employers	Patients/Employees	The state	Pension insurance scheme
Responsibilities in RTW	General responsibility for reimbursing necessary health care		Sick pay for first 2 years, reintegration of sick employees into the workplace		Sick pay after 2 years of inability to work	
Carrying the costs of RTW interventions	Reimbursement for patients with multidisciplinary rehabilitation need	Intervention costs				
Receiving the financial returns of RTW	Lower future health care costs, however, high budget impact		Fewer productivity losses, no replacement for employee needed	Ability to generate an income		Less early-retirement-pension payments
	**-**	**--**	**-**	**+**	**--**	**-**
Incentive for financing RTW for cancer patients^a^	Lower future health care costs are long-run, considerable budget impact	Carrying the costs, but not receiving financial returns.	Status quo is financially beneficial for employers.	Incentive for an acceptable out-of-pocket payment	Not receiving any financial returns	Status quo is financially beneficial.

^a^The distribution of costs and financial benefits in which the costs as well as the financial returns are incurred by the same stakeholder, incentivizes the financing and implementation of RTW. For stakeholders who receive financial benefits, but do not need to carry the costs, the current financing arrangement is very attractive. Thus, they do not have an interest in changing the financial structure. However, if they would need to take over (a part of) the financing, this would be acceptable. For stakeholders who need to carry the costs, but do not receive financial returns, an incentive to finance RTW does not exist, as they it will only cost them

#### EU-countries

**Fig. 4:**
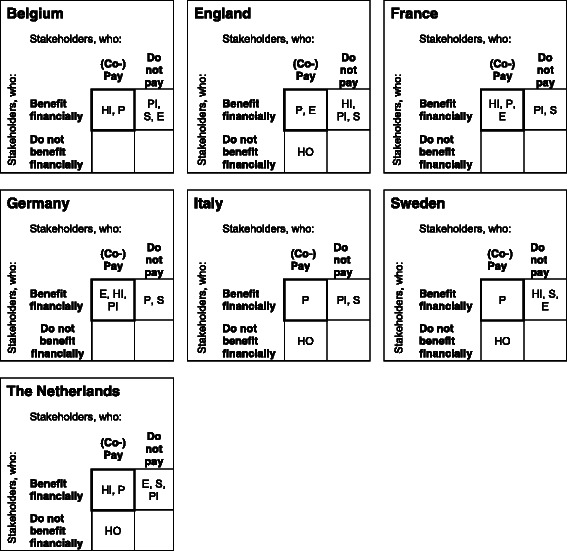
Incentive in EU-countries for financing RTW for cancer patients. The stakeholders placed in the framed square have a financial incentive for financing RTW interventions. The more stakeholders are placed in the framed square, the greater the incentives for implementing RTW are. If this is the patient/the employee, this is less beneficial then when this is another stakeholder, as it is not feasible that the patients carries the costs for the intervention alone. HI = Health insurance, HO = Hospital or health care provider, E = Employer, S = State, PI = Pension insurance scheme, P = Patient/employee

Eleven of the 40 questionnaires (28 %) that were sent to cancer centres were returned (Belgium = 1, England = 2, France = 1, Germany = 2, Italy = 3, Sweden = 1, The Netherlands = 1). Respondents included researchers, scientific directors, medical directors, a director of the psychosocial service, an HR-manager, and a social worker. In four of the six other EU-countries included in this analysis, similar misalignments of costs and financial benefits as in the Netherlands were observed, as shown in Fig. 4. The most beneficial situation for implementation of RTW-interventions is found in Germany and France. In Germany, the employers, health insurance, and pension insurance have financial incentives to support RTW, by being both responsible for financing RTW and receiving its financial benefits. In France this applies to employers and health insurers. In Belgium, the National Health Service in England, and the Netherlands patients/employees and one other stakeholder pay for and gain from RTW, while in Italy and Sweden the patients are the only stakeholders mentioned in both categories.

## Discussion

From the Dutch societal perspective, the BI of the RTW-intervention for cancer survivors is negative, i.e. the RTW-intervention yields more financial benefits than it costs. The BI for cancer centres is high, as these mainly shoulder the costs of providing the intervention. The way in which costs and financial benefits in the Netherlands are allocated, leads to a disincentive to offer RTW-interventions for cancer survivors. Of the six countries included in the European comparison, only Germany and France provide a payment structure that rewards the provision of RTW by health care providers.

In order to reduce the misalignment of costs and financial benefits in and outside the healthcare system and facilitate larger scale patient access, the payment and reimbursement structures need adjustment. For many countries, a more sustainable way of financing RTW may include shifting a larger share of the costs to employers or pension schemes, which primarily benefit financially from RTW-interventions. Alternatively within the current financing system, the intervention could be prescribed more selectively to patients at highest risk of not returning to work [18–20]. In addition, the counselling by the occupational physician could possibly be prescribed as a mono-dimensional intervention, when this matches with the individual patient’s need. As the costs for the counselling make up 37 % of the intervention costs, this would decrease the health care costs considerably for all stakeholders involved.

This study has some limitations; first, the potential overall health benefits of exercise programs, beyond returning to work, that may lead to lower future health care resource use are not included in the analysis [19]. Thus, the cost savings of RTW-interventions are probably underestimated. Welfare benefits that are influenced by RTW, such as sick pay, and invalidity and retirement pension, have not been included due to a lack of data. This might also lead to an underestimation of the potential cost savings and precludes a quantification of the financial benefits of RTW to the state. Finally, while a healthcare system perspective is recommended for BI analysis [7], we deviated from this recommendation to show the relation between the intervention costs and the productivity gains that extend beyond the health care system.

Regarding transferability of the costs of the Netherlands to other countries, it can be noted that the intervention costs mainly consist of labor costs and thus depend on the income level in the respective country. The number of patients who follow the intervention is a product of cancer incidence, the percentage of eligible patients, and capacity for treating patients. These would need to be adjusted to the respective country as well.

Moreover, the limitations of the data that was used for the productivity benefits which were derived from the study by Thijs et al. [18] need to be mentioned. First, given the evidence for the effectiveness of exercise it was considered unethical to randomize patients. Thus, instead, an age-matched control group recruited in another hospital was used. Still, the baseline characteristics of both groups were comparable. Second, most of the participants, around 70 %, were breast cancer patients and around 80 % were female. This is an issue in cancer survivorship research in general [20] but yields questions about the generalizability of the outcome. As breast cancer patients often are relatively young and have good treatment outcomes, they participate in intervention research more often than other groups. However, the criteria for being eligible for the intervention included treatment with curative intent and a treatment outcome that is sufficient for being able to return to work. Thus, this also is a selected group of cancer survivors, of which many might be breast cancer survivors, as these patients would fulfil these criteria more often than e.g. lung cancer patients. Therefore, and given the robustness of our findings against alternative effectiveness inputs (i.e. 5 times smaller), we consider it safe to conclude that the intervention is cost-saving for the general group of cancer survivors who are eligible for multidisciplinary return-to-work interventions.

In order to increase patient access to RTW-interventions, a consensus among stakeholders on how to arrange the financing of RTW-interventions needs to be found when the value of RTW-interventions is sufficiently demonstrated. For this purpose, more research is needed that assesses the effectiveness of RTW, and on the subgroups of patients who would benefit the most. Moreover, the value of the intangible benefits for the stakeholders and the intervention’s indirect benefits would need to be investigated to support this process.

## Conclusions

This study analysed the BI of a multidisciplinary RTW-intervention for cancer survivors and explored the allocation of the costs and financial benefits of RTW across the stakeholders involved in six EU-countries. From the Dutch societal perspective, the productivity gains of the RTW-intervention outweigh the intervention costs by far. However, the total healthcare costs are considerable and shouldered almost exclusively by health care providers. Therefore, the BI of RTW for cancer centres is very high and the current financing system does not provide the appropriate incentives for implementing RTW on a larger scale. A similar misalignment of financial incentives exists in other EU-countries, with only Germany and France providing an incentive for stakeholders to pay for RTW. To ensure patient access to RTW-programs, future investigations into the real-world effectiveness and societal impact of RTW-programs for cancer survivors are needed, as well as a consensus on how to fix the current financial misalignment.

## Abbreviations

BI: budget impact
RTW: return-to-work
